# Experiments

**Published:** 1800-07

**Authors:** Charles Darwin


### *Experiments, by Dr.* Charles Darwin.

As the attention of medical practitioners has been for some time directed to the treatment and diagnosis of Phthisis Pulmonalis; and as the distinction of the complaint, and its several stages, depend in a considerable degree upon the matter expectorated, we believe our readers will be gratified by the following Extracts from the scarce work, Dr. Charles Darwin's *Experiments establishing a Criterion between mucilaginous and purulent Matter.* An additional motive for reviving the subject at this time, is the great improvement which has been made made in Chemiſtry ſince the year 1780, when that work was publiſhed; and the hope that ſome of our Correſpondents will be induced to favour the Public with their ſentiments on the diſtinction between aerated pus and the matter of tubercules.

EXPERIMENT I. To a drachm of very fœtid *purulent matter*, obtained from a common abſceſs, fifty drops of *vitriolic acid* were added. The acid ſunk to the bottom, while the purulent matter kept in the upper part of the glaſs; but, on the addition of about two ounces of water, the whole formed a turbid white *uniform mixture*.

EXP. II. About fifty drops of *vitriolic acid* were added to a drachm of *mucus*, coughed up from the lungs, and diſcharged from the noſe; the acid ſoon diſſolved the greateſt part of the mucus, but, on a proportion of water being added to the mixture, the mucus, which had been diſſolved, *ſwam on the top* of the fluid.

EXP. III. To fifty drops of *vitriolic acid*, diluted with about an ounce of water, were added about two drachms of pure *mucus*, coughed up from the lungs, and collected from the noſe; notwithſtanding repeated agitation, they could not be made to unite;—almoſt the whole mucus continued *afloat at the top*; and the watery fluid below loſt very little of its tranſparency, acquiring only a ſlight bluiſh caſt.

EXP. IV. On the addition of about two drachms of fœtid *purulent matter*, to fifty drops of *vitriolic acid*, diluted with about an ounce of water, an *uniform turbid fluid* of a whitiſh colour was formed, and which exactly reſembled that mentioned in the firſt experiment.

EXP. V. About two drachms of very fœtid *purulent matter* were combined by agitation with as much *mucus*. Theſe two fluids were ſo blended together, that no difference could be perceived. To this was added a mixture of fifty drops of *vitriolic acid* with two ounces of water. In a ſhort time the fluid in the under part of the glaſs had the ſame turbid appearance as in Experiment firſt and fourth, while the mucus apparently deprived of purulent matter ſwam on the top.

EXP. VI. All the five glaſſes mentioned in the preceding experiments, were allowed to remain at reſt about half an hour, during that time the glaſſes mentioned in experiments ſecond and third, which contained the mucus, remained unchanged. But the firſt, fourth, and fifth depoſited each a white coloured ſediment in conſiderable quantity; the fluid above the ſediment in firſt and fourth had become ſomewhat more tranſparent, but ſtill continued of a white colour. In the fifth the mucus continued to float on the ſurface as before

Exp. VII. The *mucus* collected from the top of the fifth was put into another glafs, and an ounce of water impregnated with fifty drops of *cauftic alkaline* lixivium * was added; but *no union* or folution could be produced even by the aid of triture.

Exp. VIII. Fifty drops of the *alkaline lixivium* were added to each of the phials No. 1, 2, 3, and 4; when 1 and 4 were agitated, the white fediment was again diffufed in them; but, in a fhort time it fubfided, leaving the liquor above of the fame whitifh colour as before the addition; the agitation of 2 and 3 had no effect in producing any union farther than before, the mucus in both ftill continuing to float on the furface.

Exp. IX. The *mucus* ufed in 7 and 8 was again put into a glafs by itfelf, and about two drachms of *cauftic alkaline* lixivium added to it. In this, by the aid of agitation, it was foon *completely diffolved*, lofing entirely its vifcidity, and leaving a froth on the top.

Exp. X. Two ounces of water added to the folution of *mucus in alkali*, No. 9, produced no decompofition; and had the effect only of giving the fluid a greater tranfparency; no change was produced on this fluid, when allowed to remain at reft for the fpace of half an hour, except that the froth on the furface difappeared.

Exp. XI. To half the liquor of 10, about a drachm of *nitrous acid* was added; the mixture became of a more turbid and white colour, but no depofition took place.

Exp. XII. A fimilar change of colour took place on the addition of a certain quantity of the *muriatic acid* to the remaining half of No. 10. But, even after the mixture had ftood for half an hour, no fediment had fallen to the bottom of the veffel.

Exp. XIII. When the white coloured liquor on the furface was poured off from No. 1, 4, and 5, each of which depofited a fediment refembling *pus*, the fediments were again obferved to emit a very fœtid fmell. To thefe fediments, collected in one glafs, about two drachms of *nitrous acid* were added. The mixture for a little time appeared uniform, but in a few minutes a thick white cloud was obferved on the top of it, the liquor below being of a wheyifh colour.

Exp. XIV. To the liquor, No. 13, was added about two ounces of water: The cloud which before fwam on the furface, was, on flight agitation, uniformly diffufed through it; and when the mixture was allowed to remain at reft for a fhort time, a fediment

* Pharmacop. Edinens.

fediment fell to the bottom, exactly refembling that which had appeared in Obfervation 6, to have happened in Experiment 1, 4, and 5; and this fediment, when the fluid above was poured off, had alfo a fœtid fmell.

[ To be continued. ]

